# Pneumococcal population genomics changes during the early time period of conjugate vaccine uptake in southern India

**DOI:** 10.1099/mgen.0.001191

**Published:** 2024-02-05

**Authors:** Iftekhar M. Rafiqullah, Rosemol Varghese, K. Taylor Hellmann, Aravind Velmurugan, Ayyanraj Neeravi, Jones Lionel Kumar Daniel, Jorge E. Vidal, Rajeev Z. Kompithra, Valsan P. Verghese, Balaji Veeraraghavan, D. Ashley Robinson

**Affiliations:** ^1^​ Department of Cell and Molecular Biology, University of Mississippi Medical Center, Jackson, MS, USA; ^2^​ Department of Clinical Microbiology, Christian Medical College and Hospital, Vellore, India; ^3^​ Center for Immunology and Microbial Research, University of Mississippi Medical Center, Jackson, MS, USA; ^4^​ Department of Child Health, Christian Medical College and Hospital, Vellore, India

**Keywords:** *Streptococcus pneumoniae*, population genomics, vaccines, antimicrobial resistance, recombination

## Abstract

*Streptococcus pneumoniae* is a major cause of invasive disease of young children in low- and middle-income countries. In southern India, pneumococcal conjugate vaccines (PCVs) that can prevent invasive pneumococcal disease began to be used more frequently after 2015. To characterize pneumococcal evolution during the early time period of PCV uptake in southern India, genomes were sequenced and selected characteristics were determined for 402 invasive isolates collected from children <5 years of age during routine surveillance from 1991 to 2020. Overall, the prevalence and diversity of vaccine type (VT) and non-vaccine type (NVT) isolates did not significantly change post-uptake of PCV. Individually, serotype 1 and global pneumococcal sequence cluster (GPSC or strain lineage) 2 significantly decreased, whereas serotypes 6B, 9V and 19A and GPSCs 1, 6, 10 and 23 significantly increased in proportion post-uptake of PCV. Resistance determinants to penicillin, erythromycin, co-trimoxazole, fluoroquinolones and tetracycline, and multidrug resistance significantly increased in proportion post-uptake of PCV and especially among VT isolates. Co-trimoxazole resistance determinants were common pre- and post-uptake of PCV (85 and 93 %, respectively) and experienced the highest rates of recombination in the genome. Accessory gene frequencies were seen to be changing by small amounts across the frequency spectrum specifically among VT isolates, with the largest changes linked to antimicrobial resistance determinants. In summary, these results indicate that as of 2020 this pneumococcal population was not yet approaching a PCV-induced equilibrium and they highlight changes related to antimicrobial resistance. Augmenting PCV coverage and prudent use of antimicrobials are needed to counter invasive pneumococcal disease in this region.

## Data Summary

Sequencing reads are deposited in the European Nucleotide Archive with study accession PRJEB47847. Sample accessions are listed in Table S1, available in the online version of this article. C source code for calculating bootstrapped RMSE of accessory gene frequencies by time periods is deposited in GitHub (https://github.com/IftekharUMC/PneumococcalStudy).

**Impact Statement**

This study reports on genomic changes among invasive pneumococci from young children in southern India during the early time period of conjugate vaccine uptake. Genome sequencing of a surveillance collection of invasive isolates is used to provide baseline prevalence data on capsular serotypes, strain lineages, antimicrobial resistance determinants and accessory genes, before and after the uptake of conjugate vaccine in this region. This study reveals population genomics changes related to antimicrobial resistance but not vaccine immunity, which highlights the need for continued diligence in vaccination and antibiotic stewardship in this region.

## Introduction

Worldwide, an estimated 0.3 million children <5 years of age die each year from disease caused by *Streptococcus pneumoniae* (the pneumococcus), with the highest burden in low- and middle-income countries [1, 2]. In India, an estimated 0.1 million children <5 years of age die each year from pneumococcal pneumonia alone [3]. Pneumococcal conjugate vaccines (PCVs) that can reduce the burden of serious disease were licensed for optional use in India in 2006. PCVs were included in the universal immunization programme of India in 2017, with an initial focus on the northern and central Indian states with the highest rates of death due to pneumococcal pneumonia [4]. At present, the three PCVs available in India include PCV13-Prevnar (Pfizer), PCV10-Synflorix (GlaxoSmithKline) and PCV10-Pneumosil (Serum Institute of India).

In high-income countries, the widespread use of PCVs has resulted in massive reductions of invasive pneumococcal disease caused by the targeted capsular serotypes (vaccine types; VTs) [5, 6]. However, a subsequent increase in carriage and disease from serotypes not targeted by the PCVs (non-vaccine types; NVTs) also has been observed [7–9]. These post-vaccination population changes often are attributed to serotype replacement, whereby NVTs expanded in the ecological niche vacated by VTs, and can involve serotype switching, whereby otherwise fit VT strains acquired NVT capsular genes [10, 11]. Additional post-vaccination population changes have included antimicrobial resistance [12, 13], and in the profile of accessory genes [14, 15] and other pneumococcal characteristics [16, 17].

In India, PCVs target only 10–13 out of 100 known pneumococcal serotypes, and there is heterogeneity in the geographical distribution of serotypes [18]. Furthermore, *in vivo* studies have shown that *S. pneumoniae* can vary its genome relatively quickly through recombination with co-colonizing [19] or co-infecting [20] strains and therefore potentially rapidly adapt to human interventions. Thus, immunization programmes using PCVs need to be accompanied by surveillance studies to closely monitor the efficacy of PCVs and the associated evolution of pneumococcal populations. Genomic surveillance of pneumococci from across India prior to the rollout of the universal immunization programme of 2017 has highlighted one multidrug-resistant strain lineage (global pneumococcal sequence cluster 10) that expresses VT and NVT serotypes and has potential to increase in prevalence following widespread use of PCVs [21].

**Fig. 1.**
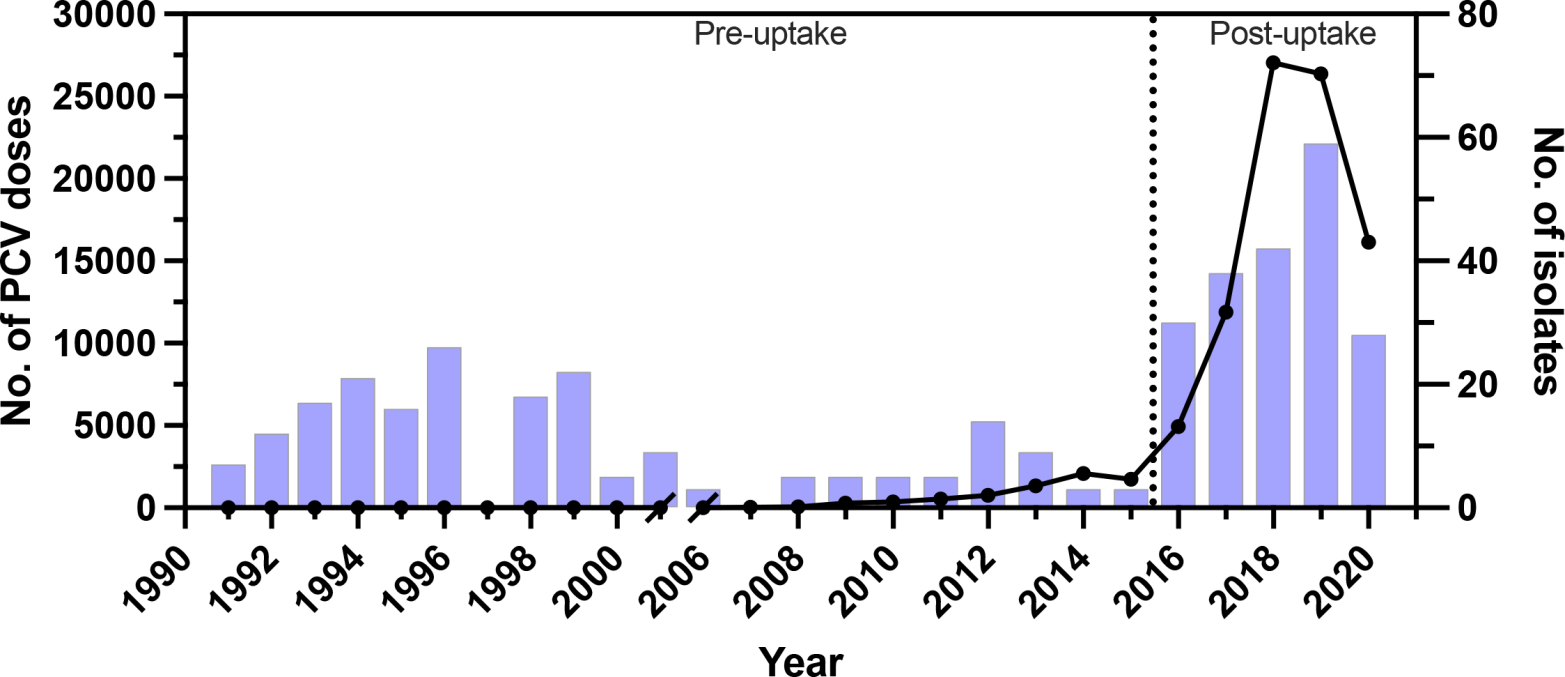
Timeline showing the number of PCV doses administered at CMC in Vellore, southern India (line), and the number of invasive isolates included in this study from surveillance of invasive pneumococcal disease in young children (bars). The delineation of PCV pre- and post-uptake time periods is based on the sustained increase in the number of PCV doses administered in the CMC immunization clinic after 2015.

Christian Medical College (CMC) in Vellore, southern India, first administered doses of PCV in 2007. According to CMC immunization records, there was a sustained uptake in the number of PCV doses administered after 2015 and disruption from the SARS-CoV-2 pandemic in 2020 (Fig. 1). Here, we report the results from genomic surveillance of invasive pneumococci from young children before and after the uptake of PCV in southern India. Population genomics changes are revealed that relate to antimicrobial resistance but not vaccine immunity. These results point to the need for continued diligence in vaccination and antibiotic stewardship in this region.

## Methods

### Bacterial isolates

The study isolates were chosen randomly among those revived from the archived isolate collection, to obtain roughly equal sample sizes before and after the uptake of PCV in this region. The delineation of PCV pre- and post-uptake time periods is based on the sustained increase in the number of PCV doses administered in the CMC immunization clinic after 2015 (Fig. 1). The specific PCV administered could be either of PCV13-Prevnar or PCV10-Synflorix, as PCV10-Pneumosil only became available throughout India by mid-2021. Besides representing a random sample from these time periods, the isolates are geographically representative because most paediatric patients reside within a radius of approximately 50 km from CMC, comprising parts of the southern states of Tamil Nadu and Andhra Pradesh [22].

A total of 402 *S*. *pneumoniae* isolates were included from invasive disease of children <5 years of age admitted to CMC (Table S1). Among these isolates, 161 were from blood, 34 were from cerebrospinal fluid, 26 were from other sterile fluid and 181 were from an unrecorded but normally sterile tissue site. Among these isolates, 205 were from the PCV pre-uptake period (1991–2015) and 197 were from the PCV post-uptake period (2016–2020) (Fig. 1).

After revival from lyophilized storage, isolates were stored in skimmed milk-glycerol medium stock at −70 °C and subcultured on 7–10 % sheep blood agar at 37 °C with 5–7 % CO_2_. The archived isolates were reconfirmed using CDC-recommended confirmatory methods such as optochin susceptibility and bile solubility. Serotype was tested by the Quellung reaction with pneumococcal antiserum (Statens Serum Institut, Denmark).

### Genome sequencing and initial bioinformatics analysis

Genomic DNA was isolated with a Promega Wizard kit (Sigma Aldrich) and concentration was checked with a Qubit Assay kit (Thermo Fisher). Construction of 150 bp paired-end libraries and genome sequencing to at least 100× coverage was done with the Illumina platform by AgriGenome Laboratories, India. Sequence reads were adapter-trimmed and filtered for minimum base quality (Q12) and minimum length [15] with bbduk v38.08 from the BBTools package [23]. Genomes were assembled *de novo* with Spades v3.11.1 [24] using a k-mer size of 75 bp. Resulting contigs were filtered for minimum length (500 bp). CheckM v1.2.0 [25] was used to filter assemblies for completeness (>95 %) and contamination (<5 %).

### Analysis of serotypes, strain lineages and antibiotic resistance determinants

The suite of bioinformatics tools available through Pathogenwatch v0.0.1 (https://pathogen.watch) were used with the assemblies to identify serotypes, strain lineages and antibiotic resistance determinants. Serotype identification used SeroBA [26]. In four of 402 (1 %) isolates where Quellung and SeroBA differed in serotype assignment within the same serogroup, the SeroBA result was used after inspection of the sequences. Strain lineage identification used PopPUNK [27] to assign global pneumococcal sequence clusters (GPSCs) and, for context, multilocus sequence typing (MLST) [28] was used to assign multilocus sequence types. Antibiotic resistance determinants, including mutations and acquired genes, were identified by blastn with a curated sequence library (https://github.com/pathogenwatch/amr-libraries). Software developed and validated by the CDC [29] was used to estimate minimum inhibitory concentrations (MICs) to penicillin. Following Nagaraj *et al*. [21], breakpoints for penicillin resistance in meningitis were used (predicted MIC≥0.12 µg ml^−1^).

### Core genome alignment and phylogenetic analysis

Pseudoreads were generated from the assemblies with samtools wgsim v0.3.1 [29] and mapped to the reference sequence of serotype 23F *S*. *pneumoniae* strain ATCC700669 (GenBank accession FM211187) with bwa mem v0.7.12 [30]. Mapped reads were processed with GenomeAnalysisToolkit v2.8–1 [31] as done previously [32] to generate a quality-filtered core genome alignment of invariant nucleotides and biallelic SNPs (biSNPs). The resulting alignment of 1 445 787 bp included 99 739 biSNPs. PhyML v3.3 [33] was used for phylogenetic analysis of the alignment with the HKY+G+I model of nucleotide substitution. The phylogeny was outgroup-rooted based on the known early-branching position of non-encapsulated *S. pneumoniae* strains of ST344 and ST448 (Bioproject accession PRJEB2340) [34].

### Recombination analysis

ClonalFrameML v1.12 [35] was used to correct the branch lengths of the phylogeny for recombination events. In addition, the number of recombination events relative to point mutations (ρ/θ) and the number of nucleotides changed by recombination events relative to point mutations (*r*/*m*, the product of ρ/θ, δ and ν) were calculated for subgroups after dropping all other isolates from the phylogeny and alignment and rerunning ClonalFrameML. The median and 95 % confidence intervals for ρ/θ and *r*/*m* were calculated from 100 parametric bootstrap values output with the emsim option. The number of recombination events affecting each nucleotide in the core genome alignment were mapped with the bedtools v2.30.0 intersect command [36].

### Accessory gene analysis

Genome assemblies were annotated with Prokka v1.13 [37] after modifying the Prodigal v2.6.3 [38] gene-calling software to allow annotation at contig edges, and gene families were subsequently identified using Roary v3.12.0 [39] with the paralogue splitting option turned off. Accessory genes (AGs) were identified as those present in 5–95 % of all isolates. This AG frequency threshold acknowledges that these are draft genome assemblies that achieved >95 % completeness. The root mean square error (RMSE) was used as a measure of average change in AG frequency through time. For this analysis, isolates were grouped based on time periods. The frequency difference between time periods of each AG was calculated, squared, then averaged across all AGs, and finally the square root returned the value to its original unit of frequency difference. The median and 95 % confidence intervals for RMSE were calculated for sample sizes of 30 isolates from 100 non-parametric bootstrap values with a C program (https://github.com/IftekharUMC/PneumococcalStudy).

### Other statistical analysis

Simpson’s diversity index and 95 % confidence intervals were calculated as described [40]. R v2.4.1 [41] was used for other statistical analysis. The mmod package [42] was used to calculate Jost’s differentiation index [43] and 95 % confidence intervals from 100 non-parametric bootstrap values. From the basic stats-package, the prop.test function was used to test for equal proportions, the cor.test function was used to calculate Pearson’s correlation coefficient and 95 % confidence intervals, and the p.adjust function was used to control the false discovery rate with the Benjamini–Hochberg procedure. Statistical significance was achieved at *P*<0.05 and trends were noted at 0.1>*P*>0.05.

## Results

### Prevalence and diversity of VT and NVT isolates by time period

Widespread use of PCVs in southern India is expected to affect the prevalence and diversity of pneumococci. To attempt to detect such population changes, pneumococcal serotypes and GPSCs (or strain lineages) were determined from the genomes of 402 invasive isolates from children <5 years of age, including 205 isolates from the PCV pre-uptake period and 197 isolates from the PCV post-uptake period (Table S1, Fig. 1).

A total of 56 capsular serotypes were identified among the isolates, with 45 serotypes from the pre-uptake period and 41 serotypes from the post-uptake period. Serotypes were classified according to their coverage by the three PCVs available in India. No significant differences were detected in the proportion of VT isolates between the pre- and post-uptake periods regardless of the PCV (Table 1). PCV13-Prevnar had the highest serotype coverage in the post-uptake period at 66 % (Table 1). Thus, in subsequent analysis, VT serotypes refer to those covered by PCV13-Prevnar, and NVT serotypes refer to those not covered by PCV13-Prevnar.

**Table 1.** Prevalence of vaccine serotypes in the PCV pre- and post-uptake time periods

	No. (%) of isolates of vaccine serotype†		
PCV*	Pre-uptake	Post-uptake	*P*‡
PCV13-Prevnar	128 (62)	130 (66)	0.523
PCV10-Synflorix	111 (54)	103 (52)	0.784
PCV10-Pneumosil	112 (55)	121 (61)	0.202
Total	205	197	

*PCV13-Prevnar (Pfizer) covers serotypes 1, 3, 4, 5, 6A, 6B, 7F, 9V, 14, 18C, 19A, 19F and 23F; PCV10-Synflorix (GSK) covers serotypes 1, 4, 5, 6B, 7F, 9V, 14, 18C, 19F and 23F; PCV10-Pneumosil (Serum Institute of India) covers serotypes 1, 5, 6A, 6B, 7F, 9V, 14, 19A, 19F and 23F.

†The pre- and post-uptake time periods were defined as in Fig. 1.

‡Test that the vaccine serotype proportion is equal in the pre- and post-uptake period.

A total of 80 GPSCs were identified among the isolates, with 55 GPSCs from the pre-uptake period and 48 GPSCs from the post-uptake period. Indices of diversity and differentiation were calculated based on the number of types and their proportions. No significant differences were detected in serotype or GPSC diversity between the pre- and post-uptake periods (Table 2). Although NVT isolates were significantly more diverse by serotype and GPSC than VT isolates in both time periods, no significant difference was detected in the differentiation of the two time periods by NVT isolates compared to VT isolates (Table 2).

**Table 2.** Diversity and differentiation of serotypes and strain lineages within and between the PCV pre- and post-uptake time periods

	Simpson’s diversity index (95 % CI) within time period†		Jost’s differentiation index (95 % CI) between time periods†
Type*	Pre-uptake	Post-uptake	Pre- vs post-uptake
Serotypes			
All	0.931 (0.908, 0.954)	0.949 (0.939, 0.960)	0.423 (0.369, 0.477)
VT	0.834 (0.784, 0.884)	0.893 (0.878, 0.908)	0.430 (0.370, 0.490)
NVT	0.964 (0.952, 0.976)	0.964 (0.950, 0.977)	0.416 (0.282, 0.550)
GPSCs			
All	0.931 (0.905, 0.956)	0.926 (0.907, 0.946)	0.591 (0.452, 0.729)
VT	0.845 (0.789, 0.901)	0.888 (0.862, 0.914)	0.636 (0.496, 0.776)
NVT	0.965 (0.952, 0.979)	0.949 (0.918, 0.980)	0.555 (0.353, 0.757)

*Vaccine type (VT) and non-vaccine type (NVT) serotypes are defined by coverage in PCV13-Prevnar. Global pneumococcal sequence clusters (GPSCs) represent strain lineages. GPSC non-assigned isolates were not included in this analysis.

†The pre- and post-uptake time periods were defined as in Fig. 1.

### Prevalence of common serotypes and GPSCs by time period

**Fig. 2.**
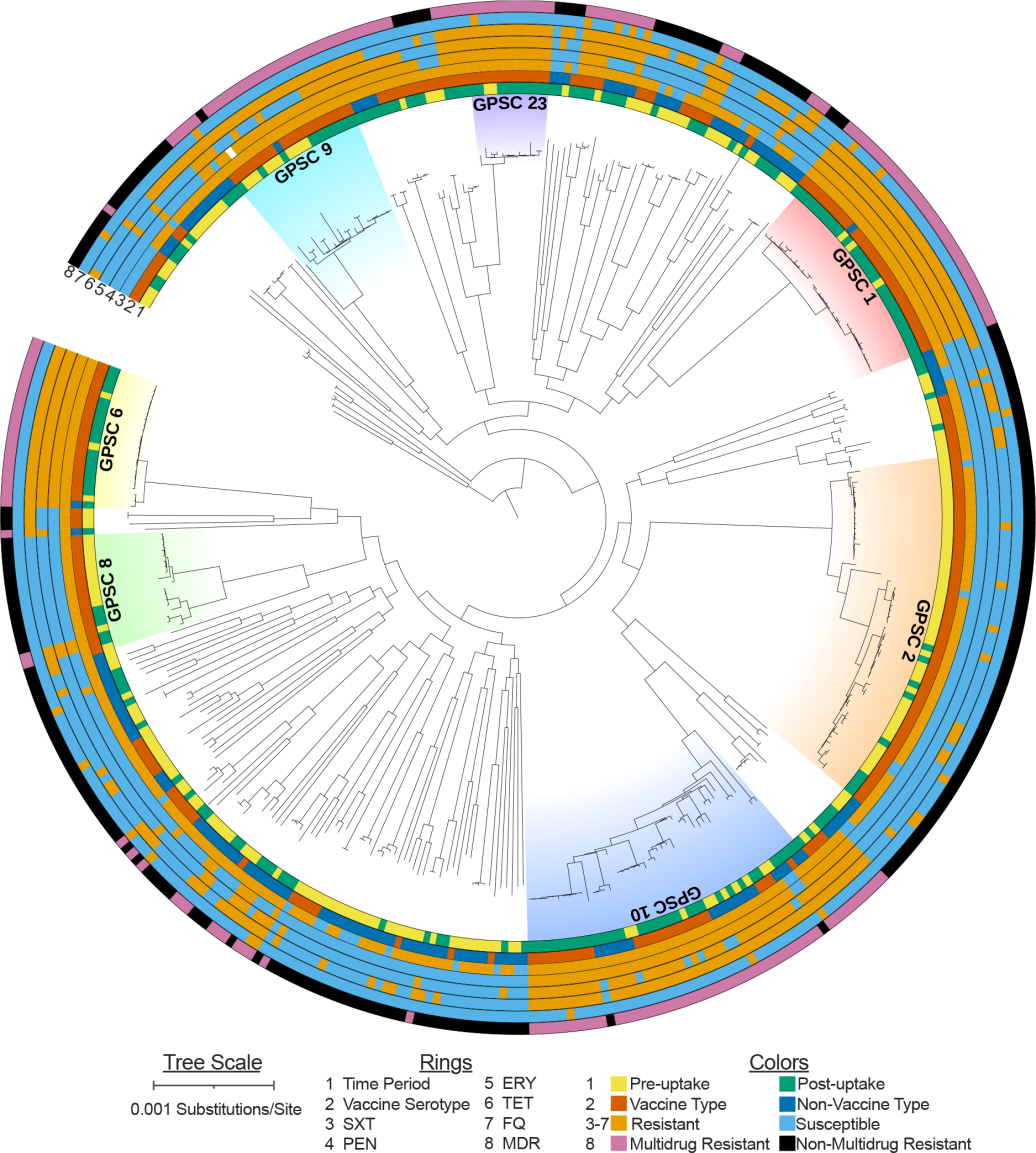
Population structure of invasive pneumococci in southern India. Maximum-likelihood tree, with branch lengths corrected for recombination events, is based on a quality-filtered core genome alignment with invariant nucleotides and biallelic SNPs. Seven prevalent GPSCs (global pneumococcal strain clusters or strain lineages) are indicated. The rings indicate isolate characteristics analysed in the text including: isolation date in the PCV pre- or post-uptake time periods as defined according to Fig. 1, vaccine type or non-vaccine type serotype defined by coverage in PCV13-Prevnar, and antimicrobial resistance determinants.

**Fig. 3.**
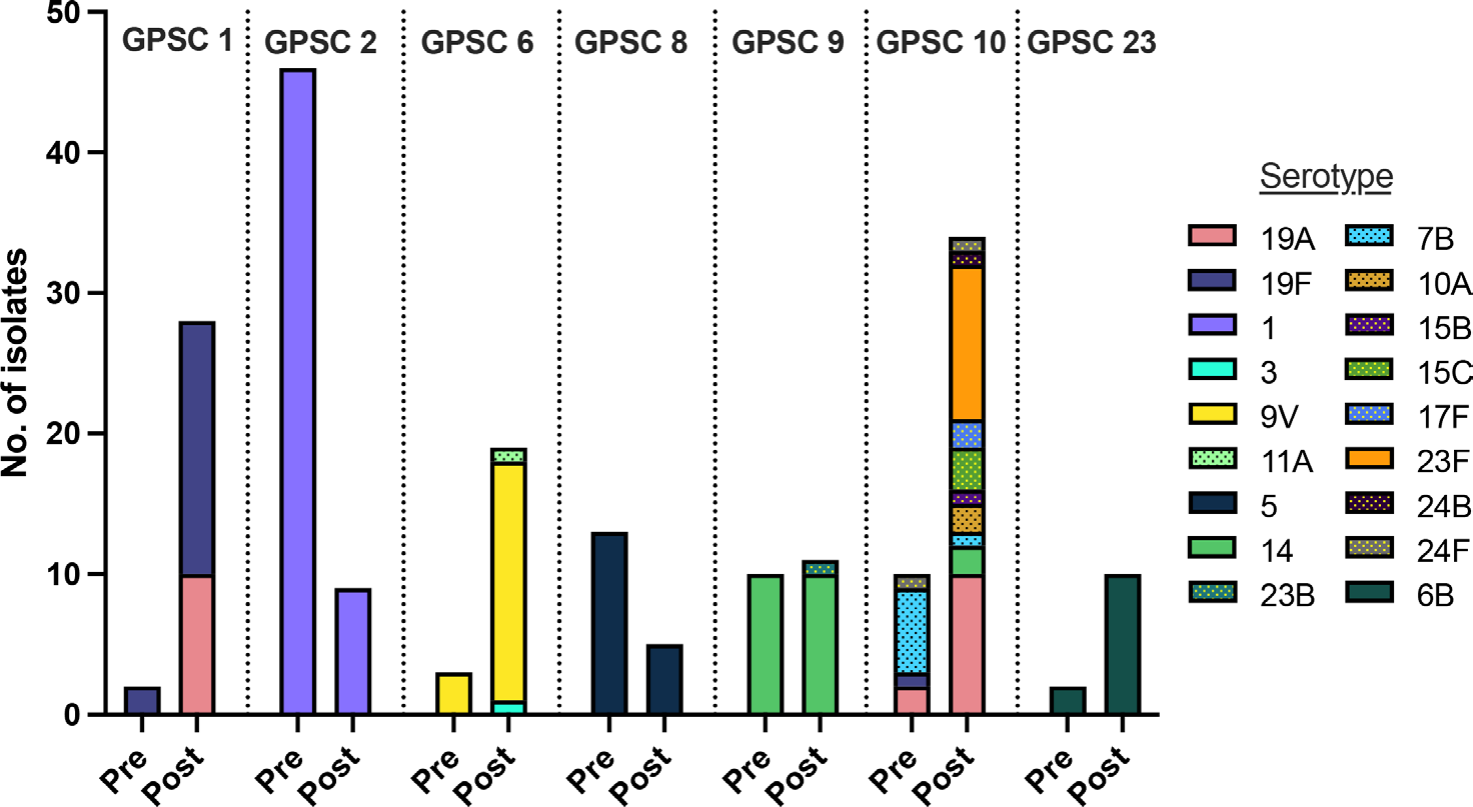
Serotype variability within the seven prevalent GPSCs (global pneumococcal strain clusters or strain lineages) in the PCV pre- and post-uptake time periods. Vaccine type (solid bars) and non-vaccine type (dotted bars) serotypes were defined by coverage in PCV13-Prevnar.

To attempt to detect changes in the prevalence of individual serotypes and GPSCs by time period, we focused on the most common types. Eight serotypes and seven GPSCs were each represented by >10 isolates (Table 3). Among these types, four serotypes and five GPSCs differed significantly in proportion between the pre- and post-uptake periods. VT serotype 1 and GPSC 2 significantly decreased, whereas VT serotypes 6B, 9V and 19A and GPSCs 1, 6, 10 and 23 significantly increased in proportion between the pre- and post-uptake periods (Table 3). The seven common GPSCs were dispersed throughout the *S. pneumoniae* phylogeny (Fig. 2). These GPSCs mostly expressed VT serotypes (Figs 2 and 3).

**Table 3.** Prevalence of the most common serotypes and strain lineages in the PCV pre- and post-uptake time periods

	No. (%) of isolates of type†		
Type*	Pre-uptake	Post-uptake	*P*‡
Serotypes			
1	46 (22)	9 (5)	3.23×10^−6^
5	13 (6)	5 (3)	0.153
6B	6 (3)	19 (10)	0.024
9V	5 (2)	17 (9)	0.024
14	10 (5)	12 (6)	0.753
19A	6 (3)	20 (10)	0.024
19F	12 (6)	18 (9)	0.329
23F	8 (4)	16 (8)	0.153
GPSCs			
1	2 (1)	28 (15)	2.29×10^−6^
2	46 (23)	9 (5)	2.29×10^−6^
6	3 (2)	19 (10)	1.23×10^−3^
8	13 (7)	5 (3)	0.125
9	10 (5)	11 (6)	0.932
10	10 (5)	34 (18)	2.33×10^−4^
23	2 (1)	10 (5)	0.048
Total	205	197	

*All types represented by >10 isolates in total are listed. Global pneumococcal sequence clusters (GPSCs) represent strain lineages.

†The pre- and post-uptake time periods were defined as in Fig. 1.

‡Test that the type proportion is equal in the pre- and post-uptake periods. *P*-values were adjusted for multiple testing within type by the Benjamini–Hochberg procedure.

In contrast, GPSC 10 comprised a large number of VT and NVT isolates, including 26 VT isolates (serotypes 14, 19A, 19F, 23F) and 18 NVT isolates (serotypes 7B, 10A, 15B, 15C, 17F, 24B, 24F) (Fig. 3). The proportion of VT isolates within GPSC 10 increased by 38 % between the pre- and post-uptake periods though this was not statistically significant. The most prevalent NVT serotypes in the post-uptake period with (non-significant) increases in prevalence by time period included 35B and 17F. The 35B isolates were from three GPSCs plus two non-assigned strain lineages, whereas the 17F isolates were from four GPSCs. In summary, only certain serotypes and GPSCs that were dominated by VT isolates showed evidence of increased prevalence by time period. As of 2020, there was no evidence for increased expansion of NVT serotypes and associated GPSCs in southern India.

### Prevalence of antimicrobial resistance determinants by time period

Together, the above results showed that the overall pneumococcal population and most of the common serotypes and GPSCs were not changing in a manner expected from widespread PCV use. In fact, most of the common serotypes and GPSCs were more prevalent in the post-uptake period despite their coverage by PCVs. Therefore, isolate characteristics other than serotype were evaluated by time period.

**Fig. 4.**
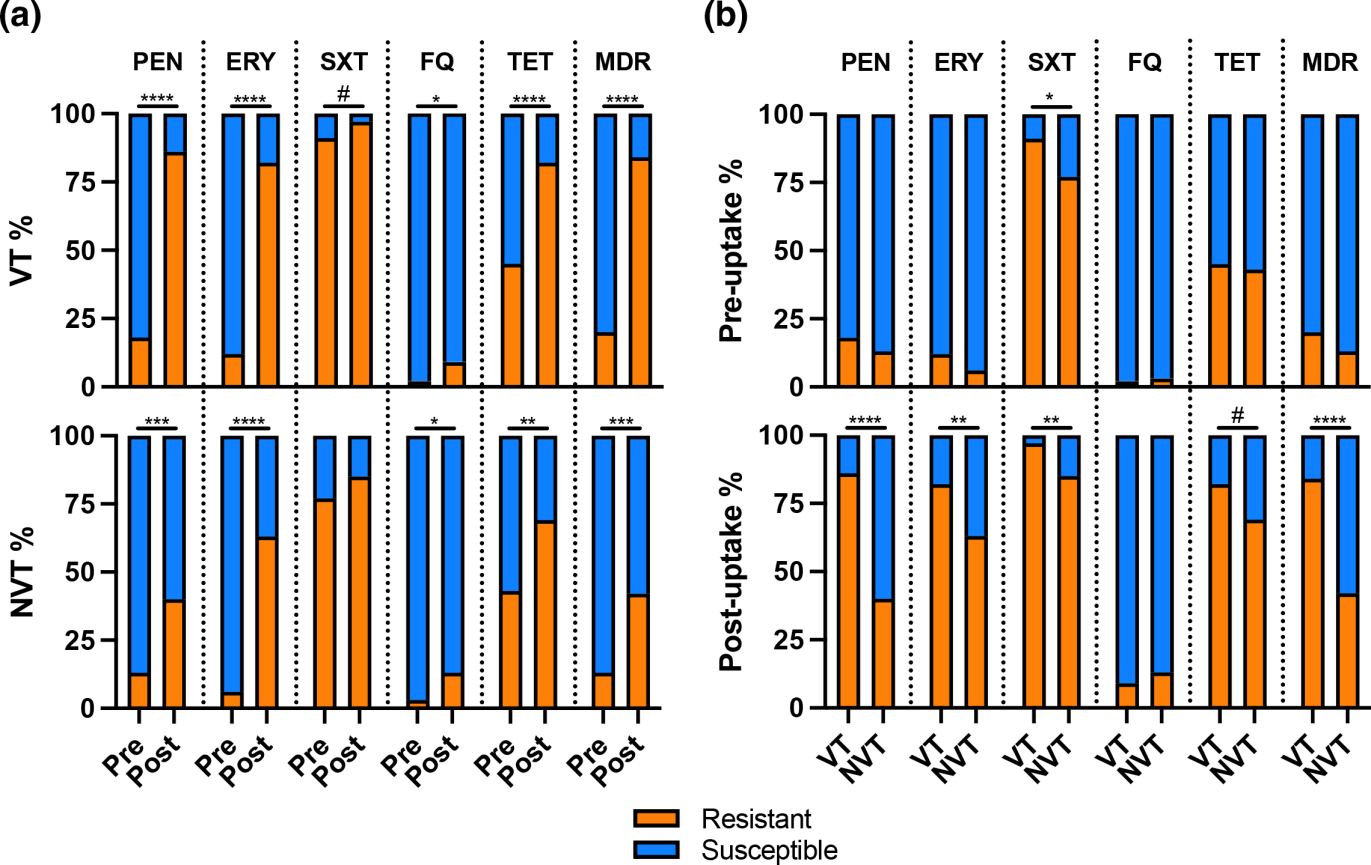
Prevalence of antimicrobial resistance determinants in the PCV pre- and post-uptake time periods according to the isolates' vaccine type (VT) or non-vaccine type (NVT) status (**a**) and vice versa (**b**). VT and NVT serotypes were defined by coverage in PCV13-Prevnar.

Resistance determinants to five classes of antimicrobials, and multidrug resistance determinants to three or more of these antimicrobials, significantly increased in proportion between the pre- and post-uptake periods (Table 4). Fluoroquinolone resistance determinants were relatively rare in both time periods (2 and 11 %, respectively), whereas co-trimoxazole resistance determinants were relatively common in both time periods (85 and 93 %, respectively) (Table 4). The other resistance determinants achieved prevalences of >70 % in the post-uptake period. Importantly, the significant increase in the proportion of resistance determinants by time period was a general phenomenon that occurred among both VT and NVT isolates (Fig. 4a), and so could not be attributed to a uniquely emerging strain lineage(s). However, the VT isolates had significantly higher proportions of resistance determinants than the NVT isolates, and this difference occurred predominantly in the post-uptake period (Fig. 4b).

**Table 4.** Prevalence of antimicrobial resistance determinants in the PCV pre- and post-uptake time periods

	No. (%) of isolates with resistance determinants†,‡		
Antimicrobial*	Pre-uptake	Post-uptake	*P*§
PEN	33 (16)	139 (71)	4.40×10^−16^
ERY	20 (10)	148 (75)	4.40×10^−16^
SXT	175 (85)	183 (93)	0.024
FQ	4 (2)	21 (11)	7.86×10^−4^
TET	90 (44)	152 (77)	2.97×10^−11^
MDR	36 (18)	148 (75)	4.40×10^−16^
Total	205	197	

*Antimicrobial resistance was predicted from genome sequences based on the presence of resistance determinants including mutations and acquired genes. PEN=penicillin, ERY=erythromycin, SXT=co-trimoxazole, FQ=fluoroquinolone, TET=tetracycline, MDR=resistant to ≥3 of these antimicrobials.

†The pre- and post-uptake time periods were defined as in Fig. 1.

‡Total number of pre-uptake isolates for PEN is 204 due to one non-assigned isolate (four intermediate and fully resistant isolates are considered as resistant for SXT).

§Test that the resistance proportion is equal in the pre- and post-uptake periods. *P*-values were adjusted for multiple testing by the Benjamini–Hochberg procedure.

### High rates of recombination affecting co-trimoxazole resistance loci

**Fig. 5.**
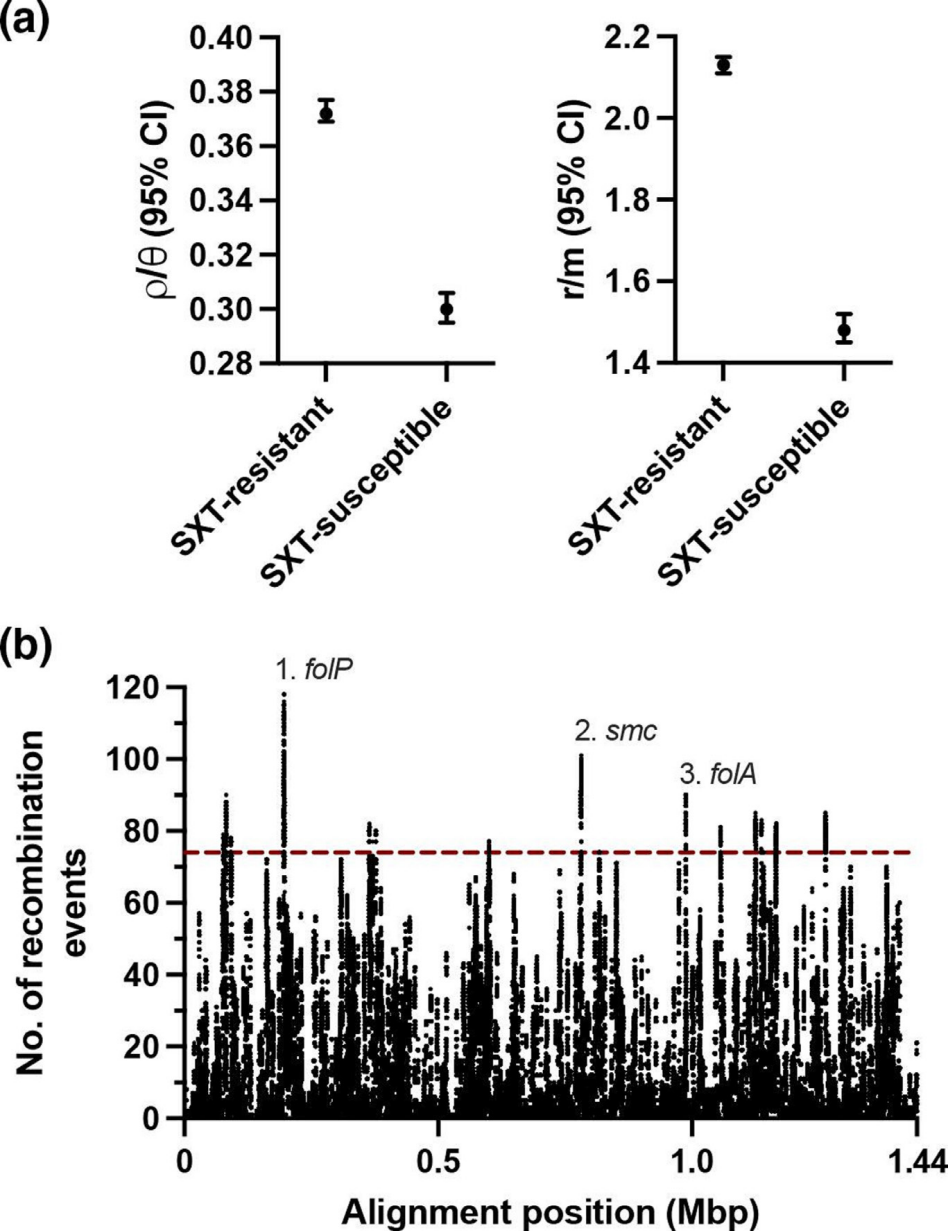
Unusually high rates of recombination at co-trimoxazole (SXT) resistance loci. The number of recombination events relative to point mutations (ρ/θ) (**a**), and the number of nucleotides changed by recombination events relative to point mutations (*r*/*m*) (**b**), among isolates with and without co-trimoxazole resistance determinants. Bars indicate 95 % confidence intervals. The number of recombination events per base pair (among base pairs with ≥1 event) in the core genome alignment. Line indicates the 99.5th percentile. The three loci with the most recombination events per base pair are indicated.

The high prevalence of resistance determinants for co-trimoxazole in both time periods suggested a history of selection at these loci. We detected significantly higher numbers of recombination events relative to point mutations (ρ/θ) and significantly higher numbers of nucleotides changed by recombination events relative to point mutations (*r*/*m*) among the isolates with co-trimoxazole resistance determinants compared to isolates without such determinants (Fig. 5a). The number of recombination events occurring across the pneumococcal chromosome were mapped to gain understanding of the rate of recombination at co-trimoxazole resistance loci compared to other loci. Remarkably, the first and third highest peaks of recombination events per nucleotide in the chromosome occurred in the *folP* and *folA* genes, respectively, which encode co-trimoxazole resistance (Fig. 5b). Insertion–deletion polymorphisms in dihydropteroate synthase encoded by *folP*, and point mutations in dihydrofolate reductase encoded by *folA*, are the genetic basis for co-trimoxazole resistance among pneumococci [44]. These results showed an unusually high rate of recombination at these two resistance loci. The second highest peak of recombination events per nucleotide occurred in the *smc* gene, which has been implicated in pneumococcal chromosome segregation [45] but, to our knowledge, has not been implicated in antimicrobial resistance.

### Accessory gene frequencies by time period

AG frequencies may be sensitive indicators of PCV-induced population changes [46]. The proposed mechanism is that the most fit and prevalent pneumococci (VT) will carry a specific profile of AGs, and that human interventions such as PCVs that alter strain frequencies in a population will select for replacement pneumococci (NVT) that have acquired these AGs [14]. To attempt to detect such population changes, gene annotation and clustering was performed. A total of 6738 gene families were identified among the isolates. Of these, 1379 were identified as AGs present with an overall frequency of 5–95 % and were retained for further analysis.

**Fig. 6.**
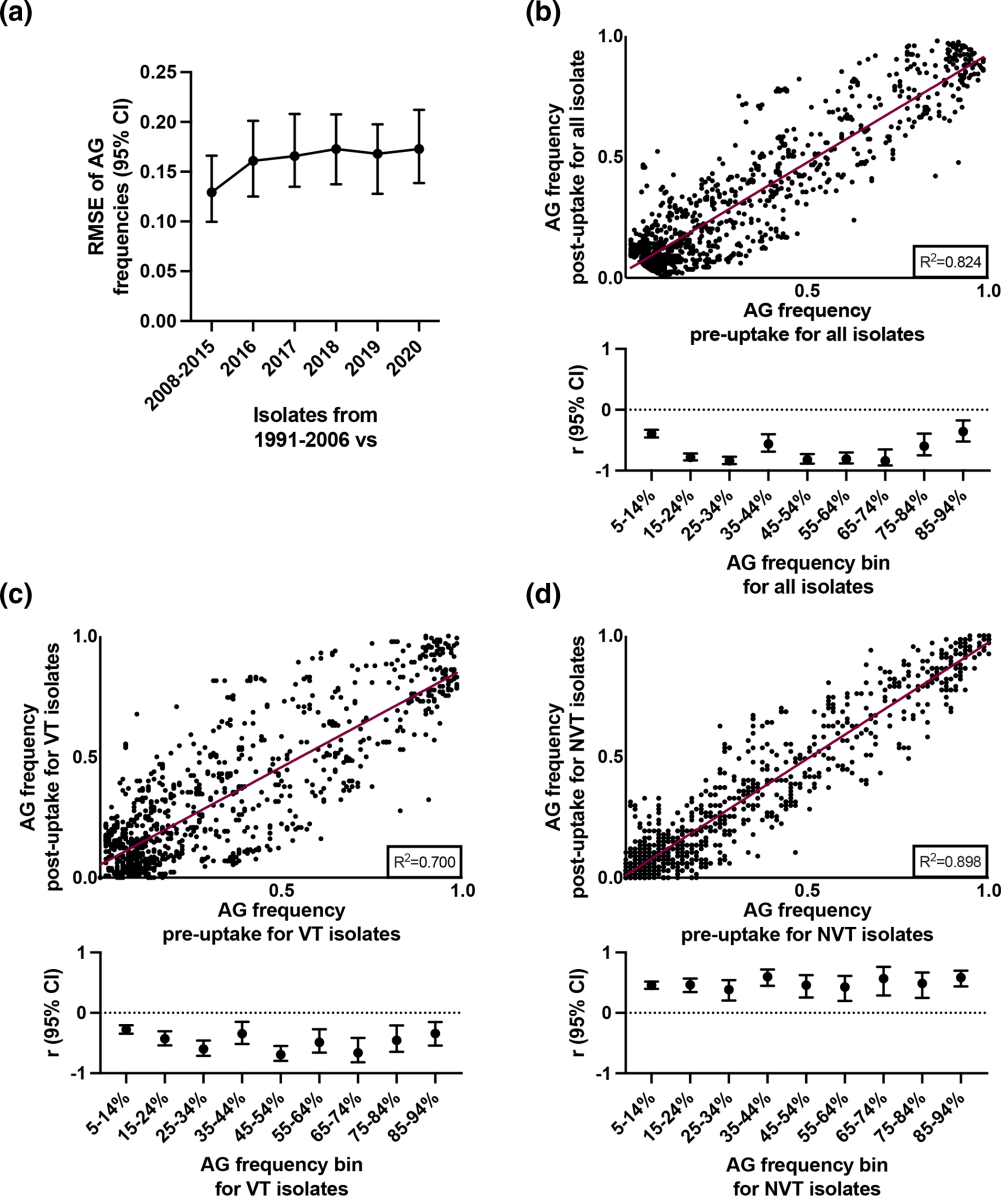
Accessory gene (AG) changes over time. Average change in AG frequency through time, using root mean square error (RMSE) to contrast frequencies from earlier versus later isolation dates (**a**). Bars indicate 95 % confidence intervals. The relationship between AG frequency in the PCV pre- vs post-uptake time periods for all isolates (**b**), vaccine type (VT) isolates (**c**) and non-vaccine type (NVT) isolates (**d**). The correlation coefficient (*r*) within 10 % frequency bins is shown below each plot, and bars indicate 95 % confidence intervals. The bins were defined from the AG frequencies among all isolates.

RMSE was used as a measure of average change in AG frequency through time. For this analysis, AG frequencies from the earliest pre-uptake isolates (1991–2006) were compared with those from the later pre-uptake isolates (2008–2015) and from each year of sampling of the post-uptake isolates (2016–2020) (Fig. 6a). While average AG frequencies from the more recent isolates were 12–17 % different from the earliest isolates, there were no significant differences among the more recent isolates (Fig. 6a). Furthermore, a significant positive correlation was observed for AG frequencies between the pre- and post-uptake periods (Fig. 6b), suggesting that rare AGs have remained rare and common AGs have remained common through time. However, when AG frequencies were binned into 10 % intervals (5–14 %, 15–24 %, etc., based on AG frequencies among all isolates) to examine small-scale changes, significant negative correlations by time period were observed for each bin (Fig. 6b, lower panel; Fig. S1). To further investigate this observation, the isolates were grouped according to VT (Fig. 6c) and NVT (Fig. 6d) status and binned as before. The significant negative correlations within bins were observed strictly among VT isolates (Fig. 6c, d, lower panels; Fig. S1). Thus, small-scale but significant changes were occurring across the AG frequency spectrum specifically among VT isolates, which reflects the earlier results that showed significant changes in prevalence by time period specifically among common VT serotypes and GPSCs (Table 3).

The three largest changes in AG frequencies between the pre- and post-uptake periods were linked to antimicrobial resistance determinants. For example, *pbp2X* alleles that confer penicillin resistance or susceptibility were mostly classified as separate AGs by the Roary software, but the frequency of resistant alleles was increasing (9–52 %) and that of susceptible alleles was decreasing (92–48 %) by time period. The frequency of a gene with homology to orf13 of Tn*916*, which is adjacent to *tetM* that confers tetracycline resistance, was also increasing (31–75 %) by time period.

## Discussion

This study has demonstrated the importance of genomic surveillance for monitoring the impact of PCVs on invasive pneumococcal populations in southern India. At CMC, PCV7 was first used in 2007 and PCV10/13 was first used in 2011, with a sustained increase in the number of administered doses after 2015. Despite the uptake of PCV, this study showed no overall changes in the prevalence or diversity of VT or NVT isolates as of 2020. This situation may be due to a relatively low population coverage of PCV, with large numbers of unvaccinated and incompletely vaccinated children. Following the rollout of the universal immunization programme of 2017 in India, the WHO estimated nationwide population coverage of PCV of 6 % in 2018, 15 % in 2019 and 21% in 2020 [47]. This level of coverage is below what has been observed in some low- and middle-income countries to result in reduced rates of VT carriage and follow-on invasive disease [48].

Although serotype 1 was significantly decreasing in prevalence post-uptake of PCV, serotypes 6B, 9V and 19A were significantly increasing in prevalence despite coverage of all three serotypes in PCV13 and coverage of serotypes 6B and 9V in both PCV10 vaccines. Serotype 19A is not covered by PCV10-Synflorix, which is the vaccine that may have been used in the majority of vaccinations in India during the study period because it is less expensive than PCV13-Prevnar and PCV10-Pneumosil was not yet available. This situation would have given serotype 19A and associated GPSCs 1 and 10 more opportunity for expansion. Both GPSCs 1 and 10 were predominantly multidrug-resistant and significantly increasing in prevalence. These results are concordant with previous studies that reported GPSC 1 (CC 320) and GPSC 10 (CC 230) as major invasive pneumococcal strains expressing multidrug resistance in India [21, 49].

Serotype replacement in a pneumococcal population following vaccination may be affected by many factors that may differ between countries [50]. In our study, no NVT serotypes showed significant increases in prevalence by time period. Nonetheless, NVT serotypes 35B and 17F warrant continued monitoring. Serotype 35B became a predominant serotype from nasopharyngeal carriage in the USA during the transition from PCV7 to PCV13 [51] and it caused increased invasive disease in South Africa after rollout of PCV13 [52]. Serotype 17F is a cause of adult invasive disease with high mortality in Denmark [53].

Post-vaccination population changes related to antimicrobial resistance also may be influenced by many factors [54]. In our study, resistance determinants were increasing among both VT and NVT isolates, but more so among the VT isolates. This is an important observation because it places emphasis on the selective pressure of antimicrobials across the pneumococcal population and not on the spread of a uniquely emerging strain lineage(s). The continued use of PCVs to counter VT serotypes in southern India brings the opportunity to remove the major source of resistance and the major source of severe pneumococcal diseases likely to require antimicrobial therapy.

Among the examined resistance determinants, those for co-trimoxazole were unique in being highly prevalent in both time periods and being recombined at unusually high rates compared to all other loci in the chromosome, suggesting an extended period of selection at these loci. A high prevalence (>75 %) of co-trimoxazole resistance has been reported previously in neighbouring countries Bangladesh and Nepal [55, 56]. Moreover, elevated rates of recombination at *folP* and/or *folA* resistance loci have been reported previously among pneumococci from southeast Asia [57, 58]. One study showed that among pneumococci from Thailand, the prevalence of resistance to penicillin, erythromycin, tetracycline and other antimicrobials was most strongly related to the duration of carriage, whereas the prevalence of resistance to co-trimoxazole was most strongly related to the rate of recombination [59]. Thus, while time of exposure to some antibiotics may be a primary driver of resistance, the rate at which strains horizontally transfer resistant alleles may be an equally important driver of resistance to co-trimoxazole. An explanation for these observations might be found in the hypothesized low fitness cost for co-trimoxazole resistance [60]. Under this hypothesis, co-trimoxazole resistance determinants may continue to spread in pneumococcal populations even after the selective pressure of the antimicrobial is removed. Thus, prudent use of this particular class of antimicrobials is especially needed in this region.

This study was well-powered to detect temporal changes between VT and NVT isolates, with approximately 200 isolates in each of the pre- and post-uptake periods. However, the sample sizes of individual serotypes and GPSCs was very low due to the overall high diversity of the population, which means the power to detect differences among individual serotypes and GPSCs was low. In addition, sampling was uneven through time with a longer period of time considered in the pre-uptake period compared to the post-uptake period. This temporal heterogeneity was examined in our analysis of accessory genes, and the largest changes occurred when comparing the earliest isolates (1991–2006) with the most recent isolates (2008–2020). Following widespread PCV vaccination in several US and European populations, large-scale changes in AG frequencies initially occurred as strains expressing VT serotypes were replaced by other strains expressing NVT serotypes [14, 15]. We found a pattern of small-scale changes occurring across the AG frequency spectrum specifically among VT isolates, which is consistent with our other results showing prevalence changes of common VT serotypes and GPSCs and their antimicrobial resistance determinants. In addition, the three largest changes in accessory gene frequency by time period were related to antimicrobial resistance.

Taken together, our results suggest that exposure to antimicrobials and not vaccines may be the primary driver of pneumococcal population changes in the early time period of PCV uptake in southern India. The study findings may serve as baseline data for this region to evaluate further changes in the pneumococcal population as PCV use increases. The study emphasizes that augmenting PCV coverage alongside prudent use of antimicrobials would together help to remove the most clinically problematic strains and also preserve treatments for the remaining strains.

## Supplementary Data

Supplementary material 1

Supplementary material 2
