# Supplementary figures and images for: Pneumococcal population genomics changes during the early time period of conjugate vaccine uptake in southern India
Source: Microb Genom. 2024 Feb 5;10(2):001191. doi: 10.1099/mgen.0.001191 (PMC10926699; doi:10.1099/mgen.0.001191)

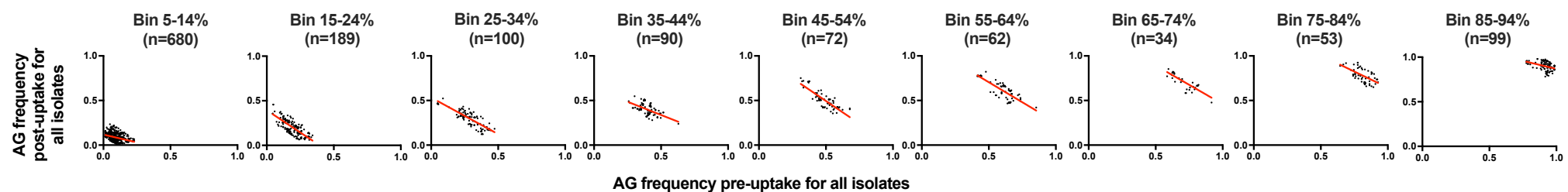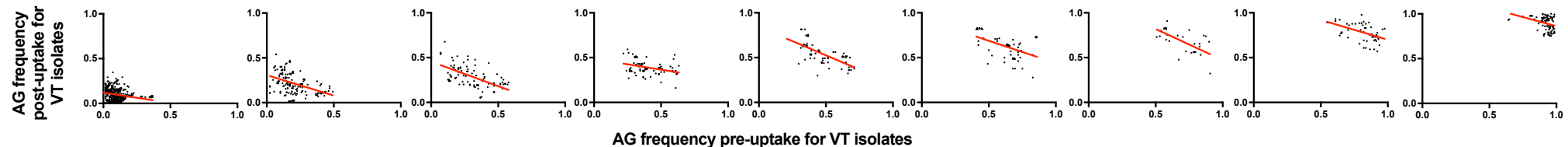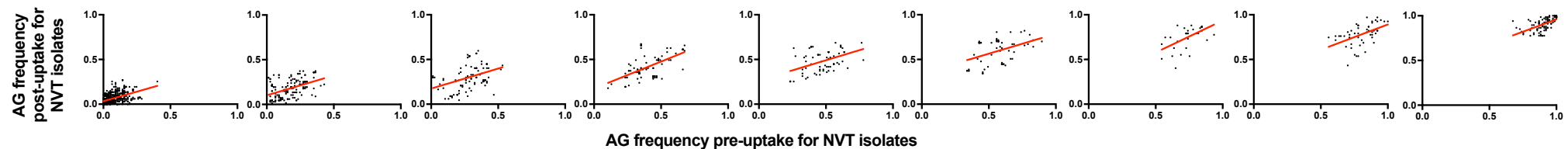

Supplement: Supplementary material 2 [file mgen-10-1191-s001.pdf]
